# Analysis of Brain Lipids in the Early-Onset Tay–Sachs Disease Mouse Model With the Combined Deficiency of β-Hexosaminidase A and Neuraminidase 3

**DOI:** 10.3389/fmolb.2022.892248

**Published:** 2022-08-08

**Authors:** Melike Can, Tugce Sengül, Secil Akyildiz Demir, Orhan K. İnci, Hande Basırlı, Volkan Seyrantepe

**Affiliations:** ^1^ Department of Molecular Biology and Genetics, Izmir Institute of Technology, Izmir, Turkey; ^2^ Izmir Institute of Technology, IYTEDEHAM, Izmir, Turkey

**Keywords:** Tay–Sachs disease, mouse model, lipidomics, brain, gangliosides

## Abstract

**Introduction:** Tay–Sachs disease is an autosomal recessively inherited lysosomal storage disease that results from loss-of-function mutations in the *HEXA* gene coding β-hexosaminidase A. *HEXA* gene deficiency affects the central nervous system owing to GM2 ganglioside accumulation in lysosomes resulting in progressive neurodegeneration in patients. We recently generated a novel mice model with a combined deficiency of β-hexosaminidase A and neuraminidase 3 (*Hexa−/−Neu3−/−*) that mimics both the neuropathological and clinical abnormalities of early-onset Tay–Sachs disease. Here, we aimed to explore the secondary accumulation of lipids in the brain of *Hexa−/−Neu3−/−* mice.

**Materials and Methods:** In the cortex and hippocampus of five-month-old *WT*, *Hexa−/−*, *Neu3−/−*, and *Hexa−/−Neu3−/−* mice, lipid levels belonging to glycerolipids, glycerophospholipids, and sterol lipids were evaluated using a shotgun lipidomics approach. The levels of myelin were also assessed by luxol fast blue staining and immunohistochemistry using antibodies against myelin basic protein. We further examined glycoconjugate and cholesterol levels by periodic acid–Schiff and filipin staining, respectively. Toluidine blue staining was also performed to display axonal degeneration.

**Results:** Among glycerophospholipids, we demonstrated elevated levels of phosphatidylcholine-ether and lysophosphatidylcholine while decreased levels of phosphatidylcholine and phosphatidylserine in both cortex and hippocampus of *Hexa−/−Neu3−/−* mice. In the glycerolipid class, we showed an alleviated level of sphingomyelin in both cortex and hippocampus, but the higher levels of diacylglycerol and triacylglycerol were detected in only the hippocampus of *Hexa−/−Neu3−/−* mice. The lower level of sterol was also detected in the cortex of *Hexa−/−Neu3−/−* mice but not in the hippocampus.

Histochemical studies showed a decrease in the myelin level and axonal degeneration indicating neuronal pathology in the brain of *Hexa−/−Neu3−/−* mice. Although glycoconjugate accumulation was evident both in the cortex and hippocampus, we did not detect any changes in the level of cholesterol.

**Conclusion:** Our results indicate that alterations in lipid metabolism and neuropathology, such as demyelination and axonal degeneration, might be related to the dysfunctionality of lipid-related cellular pathways like autophagy. Understanding of brain-specific lipid alterations contributes to evaluating the effectiveness of treatments in *Hexa−/−Neu3−/−* mice in future studies.

## Introduction

The GM2 gangliosidoses are lysosomal storage diseases that include Tay–Sachs disease (TSD), Sandhoff disease (SD), and GM2 activator deficiency (GM2AP) (Platt et al., 2012). TSD is caused by mutations in the α subunit of heterodimeric enzyme β-hexosaminidase A (HEXA), which is responsible for the conversion of GM2 to GM3 ganglioside (Yamanaka et al., 1994). Abnormal GM2 accumulation due to HEXA results in loss of motor function, seizures, developmental impairments, and death in infants with TSD (Triggs-Raine et al., 2001). There are no approved therapies, and the current standard of care is not effective at managing seizures or improving mental and motor deficits. The generation of a suitable mouse model is important for the study of TSD pathogenesis and the development of potential treatments. Unexpectedly, *Hexa−/−* mice did not show any abnormal phenotype and are clinically indistinguishable from wild-type mice until at least 1 year of age (Yamanaka et al., 1994; Phaneuf et al., 1996). Recently, a mutant mouse line depleted for both Hexa and Neu3 genes (*Hexa−/−Neu3−/−)* has been generated to understand the metabolic bypass mechanism in the ganglioside pathway. These mice showed progressive GM2 ganglioside accumulation and clinical, biochemical, and pathological abnormalities seen in Tay–Sachs patients (Seyrantepe et al., 2018). Therefore, the *Hexa−/−Neu3−/−* mice model is suitable for further investigation of disease pathologies associated with early-onset TSD.

Lipids underlie important cellular events such as cell membrane formation, cellular transport, and energy storage (Liu et al., 2021). In addition to structural roles, lipids in the nervous system have also been shown to contribute to several functions including synaptogenesis, impulse conduction, and myelin sheath maintenance (Cermenati et al., 2015). There are eight distinct subgroups of lipids; fatty acyls, glycerolipids, glycerophospholipids, sphingolipids, sterol lipids, prenol lipids, saccharolipids, and polyketides (Stephenson et al., 2017). Among them, cholesterol, sphingolipids, and glycerophospholipids are critical in maintaining homeostatic balance, neuronal membrane architecture, and proper neurodevelopment and function (Fuller and Futerman, 2018). Secondary accumulation of any of the three major classes in neurodegeneration is underscored by the number of LSDs including Gaucher disease (reviewed by Walkley and Vanier, 2009; Fuller and Futerman, 2018; Breiden and Sandhoff, 2020). In addition to cholesterol and glycosaminoglycan accumulation, which are the hallmarks of Niemann–Pick disease (NPD) and mucopolysaccharidoses (MPSs), secondary accumulation of GM2 and GM3 gangliosides also contributes to disease neuropathologies of NPD (Walkley and Vanier, 2009).

In this study, we aimed to clarify the secondary alteration of lipids in the brain of *Hexa−/−Neu3−/−* mice by shotgun lipidome analysis. We also analyzed neuropathology in the brain of *Hexa−/−Neu3−/−* mice to clarify demyelination and axonal degeneration processes using immunohistochemical and histopathological analyses.

## Method

### Animals

Single and double knock-out mouse models ( *Hexa−/−*, *Neu3−/−*, and *Hexa−/−Neu3−/−*) were generated as previously described (Seyrantepe et al., 2018). Breeding and maintenance of all mice were supplied in the Turkish Council on Animal Care (TCAC)-accredited animal facility of Izmir Institute of Technology according to the TCAC guidelines. The animal care approval was granted by the Animal Care and Use Committee of the Izmir Institute of Technology, Izmir, Turkey. Mice were housed under constant conditions (12 h light: dark cycle, room temperature 21 ± 1°C with water and food available *ad libitum*). Pups were weaned 3 weeks after birth and genotyped (Seyrantepe et al., 2018). *WT*, *Hexa−/−*, *Neu3−/−*, and *Hexa−/−Neu3−/−* mice were killed at 5 months of age.

### Shotgun Lipidomics

Five-month-old *WT*, *Hexa−/−*, *Neu3−/−*, and *Hexa−/−Neu3−/−* mice (*n* = 3) were anesthetized and perfused through the heart with 150 mM ammonium bicarbonate in water. First, the cortex and hippocampal brains were dissected and then snap-frozen in liquid nitrogen. Brain regions were homogenized twice with metal beads in 500 ul of 150 mM ammonium bicarbonate at 20 s^−1^ frequencies for 40 s. Protein concentrations of samples were detected. Lipids were determined by shotgun lipidomics analysis at Lipotype GmbH (Dresden, Germany). Briefly, a two-step chloroform/methanol procedure was used for lipid extraction (Sampaio et al., 2011). An internal lipid standard mixture including cardiolipin (CL), ceramide (Cer), diacylglycerol (DAG), hexosylceramide (HexCer), lyso-phosphatidate (LPA), lyso-phosphatidylcholine (LPC), lyso-phosphatidylethanolamine (LPE), lyso-phosphatidylglycerol (LPG), lyso-phosphatidylinositol (LPI), lyso-phosphatidylserine (LPS), phosphatidate (PA), phosphatidylcholine (PC), phosphatidylethanolamine (PE), phosphatidylglycerol (PG), phosphatidylinositol (PI), phosphatidylserine (PS), cholesterol ester (CE), sphingomyelin (SM), and triacylglycerol (TAG) was used to stimulate the samples. The obtained organic phase was transferred to an infusion plate and dried using a speed vacuum concentrator. After this process, two distinct dry extracts were collected—the first-step dry extract and the second-step dry extract. The first-step dry extract was suspended in 7.5 mM ammonium acetate in chloroform/methanol/propanol (1:2:4; V:V:V), while the second-step dry extract was suspended in a 33% ethanol solution of methylamine in chloroform/methanol (0.003:5:1; V:V:V). All these steps were performed using the Hamilton Robotics STARlet robotic platform with the Anti-Droplet Control feature for organic solvent pipetting. Samples were analyzed by direct infusion on a Q Exactive mass spectrometer (Thermo Scientific) equipped with a TriVersa NanoMate ion source (Advion Biosciences). Analysis of the samples was performed in both positive and negative ion modes with a resolution of Rm/z = 200 = 280000 for MS and Rm/z = 200 = 17500 for MS/MS experiments. MS/MS was triggered by an inclusion list encompassing corresponding MS mass ranges scanned in 1 Da increments (Surma et al., 2015). Both MS and MS/MS data were combined to monitor CE, DAG, and TAG ions as ammonium adducts; PC and PC O- as acetate adducts; and CL, PA, PE, PE O-, PG, PI, and PS as deprotonated anions. MS only was used to monitor LPA, LPE, LPE O-, LPI, and LPS as deprotonated anions; and Cer, HexCer, SM, LPC, and LPC O- as acetate adducts. Data were analyzed with the LipidXplorer (Herzog et al., 2011; Herzog et al., 2012). Lipotype Zoom was used for data post-processing and normalization. Samples having a signal-to-noise ratio >5 and a signal intensity five-fold higher than the blank samples were used for data analysis.

### Histopathology and Immunohistochemistry Analysis

Mice were deeply anesthetized (Ketamine/Ketasol mixture; 200/10 mg/kg), and then intracardiac perfusion was performed using 0.9% saline solution first and then 4% paraformaldehyde (PFA) in phosphate-buffered saline (PBS). Mouse brain samples were incubated in 4% PFA overnight and then sequentially treated with 10 and 20% sucrose solutions for 2 h followed by 30% sucrose solution overnight. Fixed samples were embedded into the Tissue-Tek OCT compound (Sakura Finetechnical, Japan) and stored at −80°C.

Fixed mouse brain samples of five-month-old *WT*, *Hexa−/−*, *Neu3−/−*, and *Hexa−/−Neu3−/−* mice were sectioned in coronal planes at 10 μm thickness using a Leica cryostat, and samples were collected on lysine-coated Histo-Bond^®^ microscope slides (Marienfeld, Germany) at −20°C. Then, they were stained with filipin (Sigma) for 2 h at room temperature followed by counterstaining with Propidium Iodide. For luxol fast blue staining (LFB), slides were stained with luxol at 60°C overnight followed by counterstaining with 0.1% cresyl violet for 15 min at room temperature. For toluidine blue staining, slides were stained with toluidine blue (Sigma) for 1.5 min at room temperature in a humidified chamber. For periodic acid–Schiff (PAS) reagent staining, slides were stained with 0.5% periodic acid for 5 min and Schiff reagent for 15 min followed by counterstaining of the slides with Gill’s hematoxylin III (Merck, Germany). All of the slides were mounted with Cytoseal 60.

In immunohistochemical analysis, coronally sectioned slides (10 μm thickness) were treated with ice-cold acetone, and then slides were blocked with blocking buffer (4% BSA, 10% goat serum, 0.3% Triton X-100, and 0.3 M glycine in PBS) for 1 h at room temperature in a humidified chamber. Anti-Mbp (1:50; Cell Signaling, Netherlands) and Anti-GM2 (1:500; KM966) were diluted in blocking buffer and applied overnight at 4°C. The goat anti-rabbit Alexa Fluor 488 secondary antibody (Abcam, United States) was used to visualize the primary antibody of anti-Mbp. The slides were mounted with Fluoroshield mounting medium with DAPI (Abcam, United States). Images were captured using a fluorescence microscope (Bx53, Olympus Corporation, Germany) equipped with a manually controlled specimen holder, a color camera, a fluorescent light source, and image analysis software (cellSens Entry, Olympus Corporation, Germany). The quantification of MBP and GM2-positive cell intensities was measured by ImageJ software (Java-based image processing program) as green color intensity separately.

### Statistical Analysis

For statistical analyses, the GraphPad QuickCalcs (GraphPad Software, United States) software was used. All the data were expressed as the mean ± SEM. The statistical differences were quantified using one-way ANOVA for all experiments. A *p*-value of less than 0.05 was determined to show statistical significance.

## Results

### Glycerophospholipid Profile in the Cortex and Hippocampus

**FIGURE 1:**
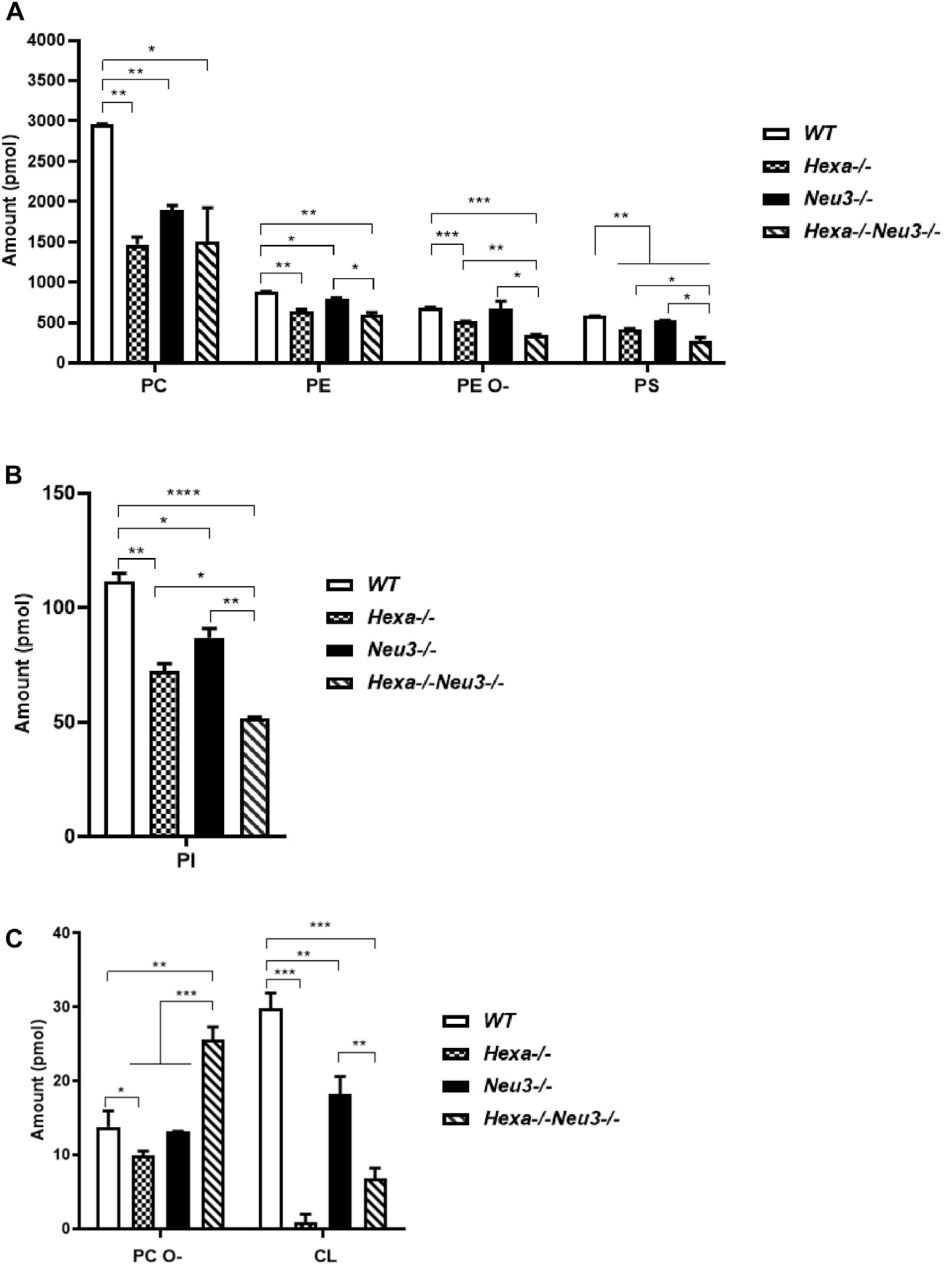
Lipidome analysis of major **(A)**, intermediate **(B)**, and minor **(C)** glycerophospholipids in the cortex of five-month-old *WT*, *Hexa−/−*, *Neu3−/−*, and *Hexa−/−Neu3−/−* mice. The data are represented as the mean ± SEM. One-way ANOVA was used for statistical analysis (*n* = 3, *p < 0.05, **p < 0.01, ***p < 0.005, and *p < 0.001).

Shotgun lipidomics analysis indicated a decreased level of PC, PE, PE O-, PS, and PI glycerophospholipids in the cortex of five-month-old *Hexa−/−*, *Neu3−/−*, and *Hexa−/−Neu3−/−* mice compared to *WT* (Figures 1A,B). We detected reduced levels of PE O-, PS, and PI glycerophospholipids in *Hexa−/−Neu3−/−* mice compared to *Hexa−/−* and *Neu3−/−* mice (Figures 1A–C). In addition, the levels of PE and CL decreased in the cortex of *Hexa−/−Neu3−/−* mice compared to *Neu3−/−* mice (Figures 1A,C). Moreover, there are significantly increased levels of PC O- in *Hexa−/−Neu3−/−* mice compared to other genotypes in the cortex (Figure 1C). Although several glycerophospholipids have distinct patterns among genotypes, no significant differences were detected for LPA, LPE, PLS, PLI, and LPE O- glycerophospholipids in the cortex (Supplementary Figure S1).

**FIGURE 2:**
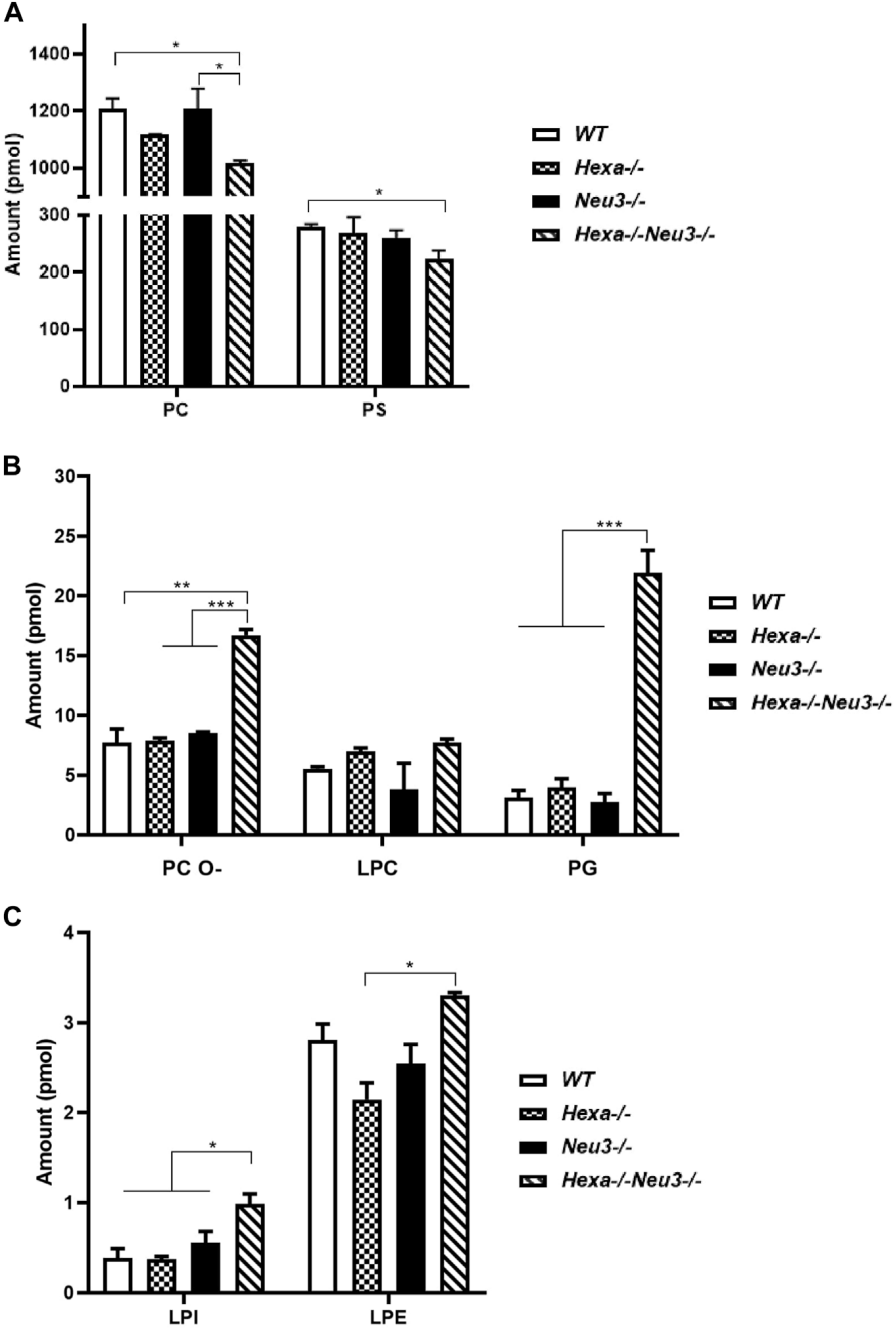
Lipidome analysis of major **(A)**, intermediate **(B)**, and minor **(C)** glycerophospholipids in the hippocampus of five-month-old *WT*, *Hexa−/−*, *Neu3−/−*, and *Hexa−/−Neu3−/−* mice. The data are represented as the mean ± SEM. One-way ANOVA was used for statistical analysis (*n* = 3, *p < 0.05, **p < 0.01, and ***p < 0.005).

In the hippocampus, the level of PC glycerophospholipid decreased in five-month-old *Hexa−/−Neu3−/−* mice compared to *WT* and *Neu3−/−* mice, while the level of PS reduced in five-month-old *Hexa−/−Neu3−/−* mice compared to *WT* (Figure 2A). Although LPC did not show any significant difference in the hippocampus of five-month-old *Hexa−/−Neu3−/−* mice, we detected elevated levels of PC O- and PG glycerophospholipids in *Hexa−/−Neu3−/−* mice compared to other genotypes (Figure 2B). Furthermore, shotgun lipidomics analysis indicated elevations of LPI in the hippocampus of *Hexa−/−Neu3−/−* mice compared to *WT*, *Hexa−/−*, and *Neu3−/−* mice (Figure 2C). We also showed that the level of LPE was significantly increased in five-month-old *Hexa−/−Neu3−/−* mice compared to *Hexa−/−* mice (Figure 2C). Other glycerophospholipids such as PE, PE O-, HexCer, PI, PA, CL, LPC O-, LPS, LPE O-, and LPA did not show significant changes in the hippocampus of five-month-old *Hexa−/−Neu3−/−* mice (Supplementary Figure S2).

### Glycerolipid Profile in the Cortex and Hippocampus

**FIGURE 3:**
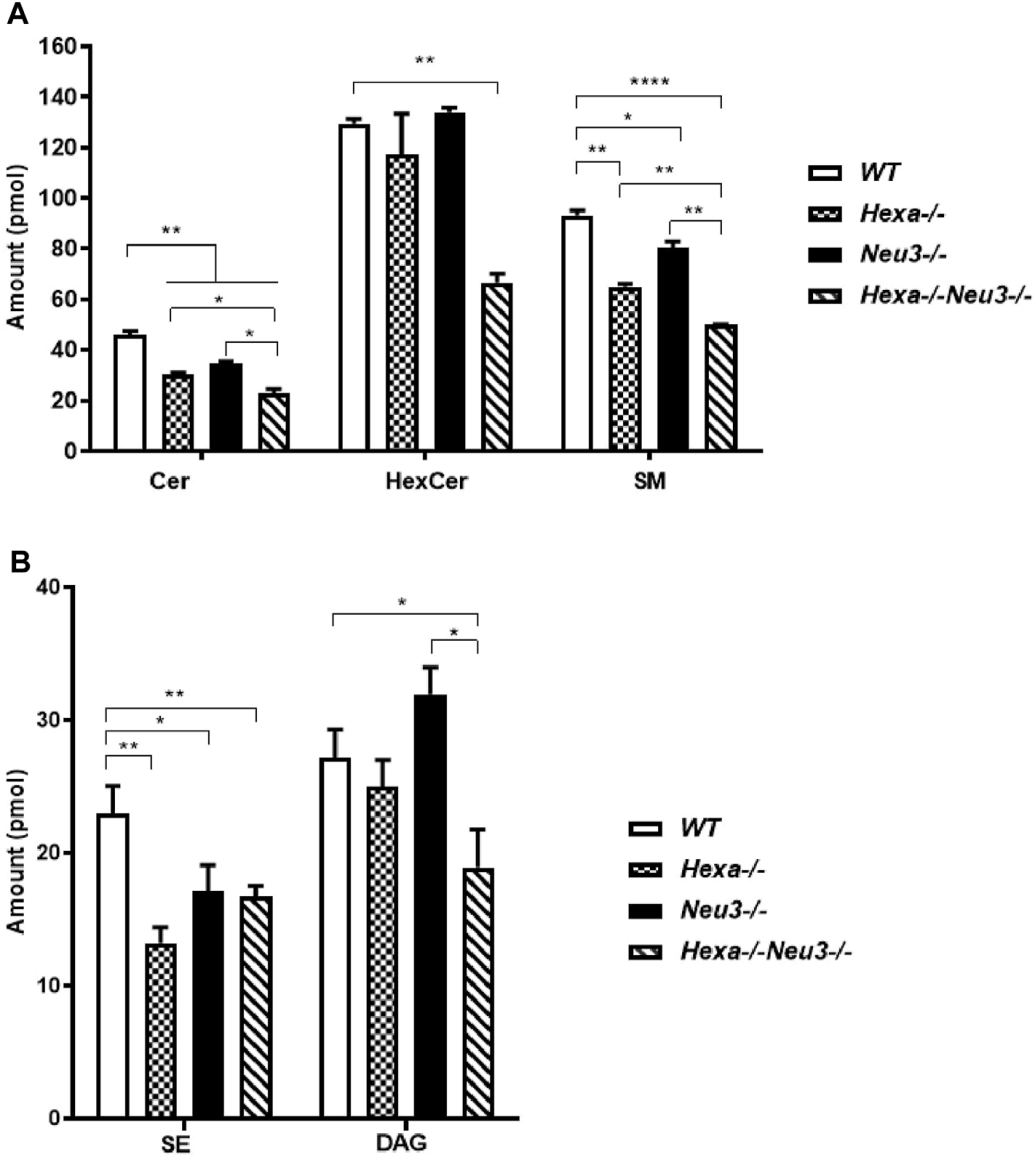
Lipidome analysis of major **(A)** and intermediate **(B)** glycerolipids in the cortex of five-month-old *WT*, *Hexa−/−*, *Neu3−/−*, and *Hexa−/−Neu3−/−* mice. The data are represented as the mean ± SEM. One-way ANOVA was used for statistical analysis (*n* = 3, *p < 0.05, **p < 0.01 and ****p < 0.001).

Lipidomics analysis was also performed to determine fluctuations of glycerolipids. Five-month-old *Hexa−/−Neu3−/−* mice displayed reduced levels of Cer and SM compared to *WT* and *Hexa−/−*, and *Neu3−/−* mice in the cortex (Figure 3A). We detected decreased levels of HexCer and DAG in the cortex of *Hexa−/−Neu3−/−* mice compared to *WT* (Figures 3A,B).

**FIGURE 4:**
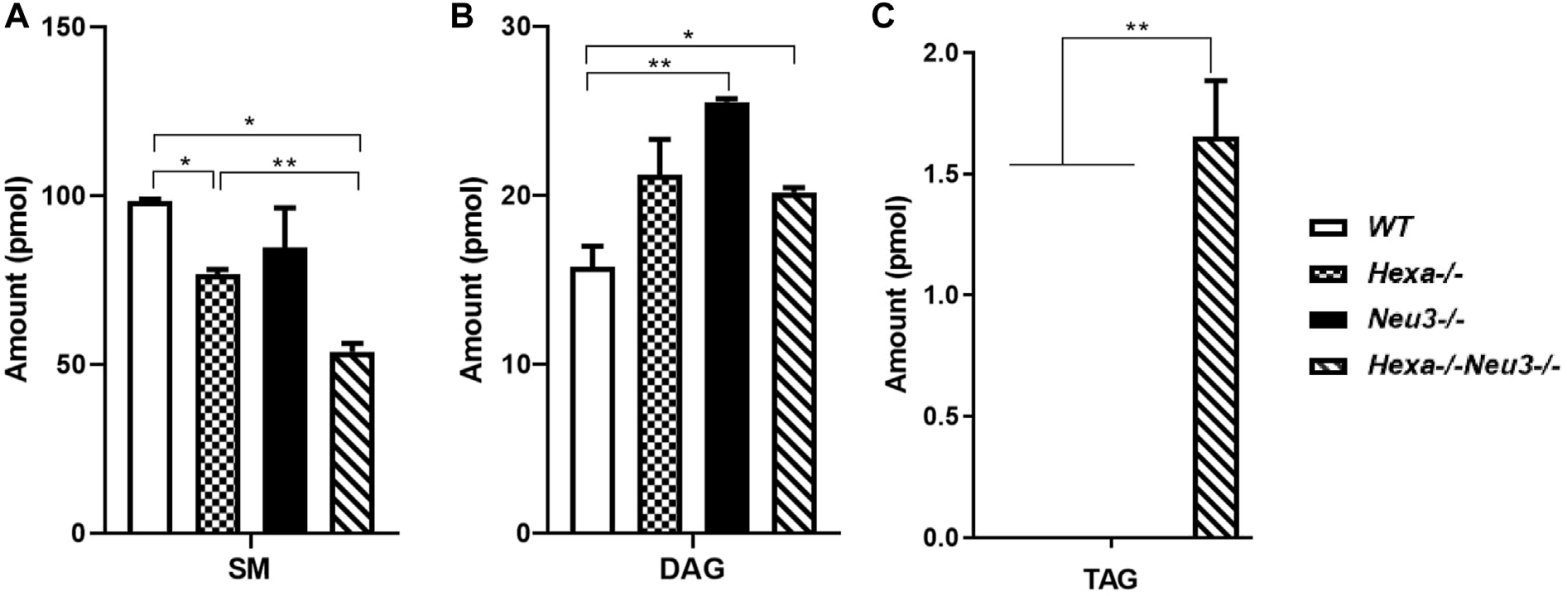
Lipidome analysis of major **(A)**, intermediate **(B)**, and minor **(C)** glycerolipids in the hippocampus of five-month-old *WT*, *Hexa−/−*, *Neu3−/−*, and *Hexa−/−Neu3−/−* mice. The data are represented as the mean ± SEM. One-way ANOVA was used for statistical analysis (*n* = 3, *p < 0.05 and **p < 0.01).

**FIGURE 5:**
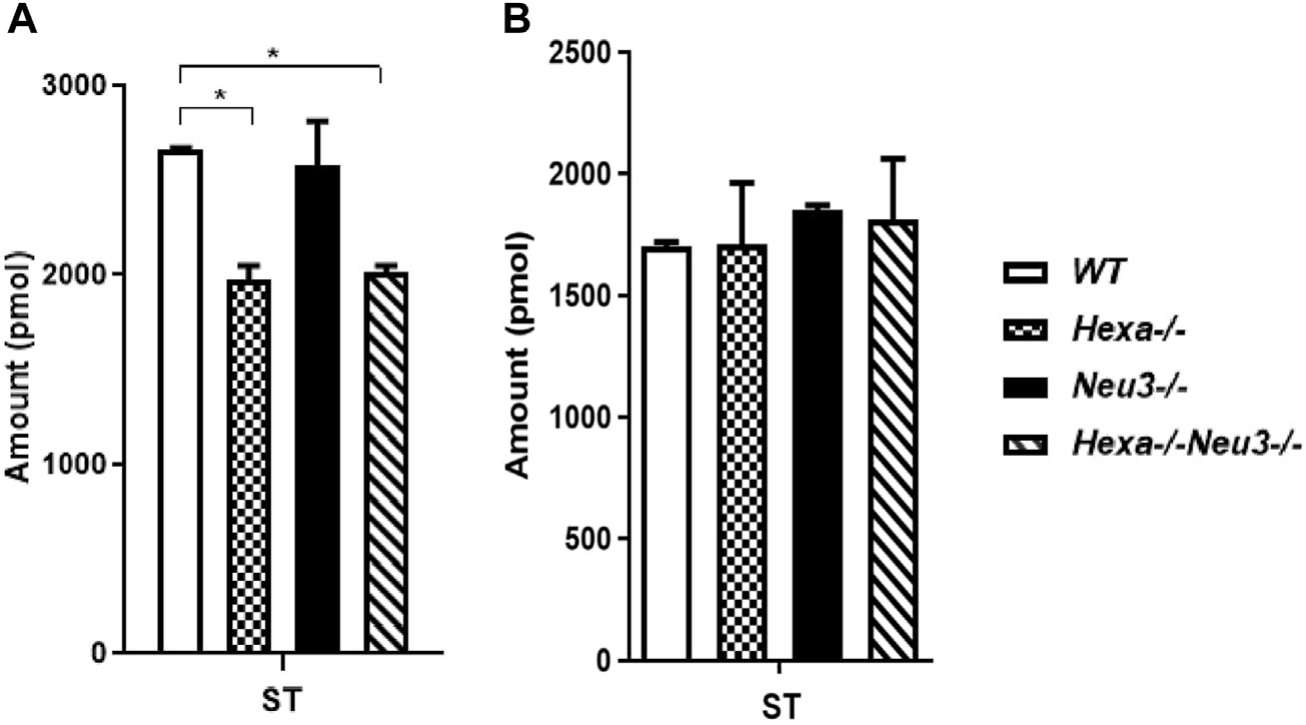
Lipidome analysis of sterol lipid (ST) in the cortex **(A)** and hippocampus **(B)** of five-month-old WT, *Hexa−/−*, *Neu3−/−*, and *Hexa−/−Neu3−/−* mice. The data are represented as the mean ± SEM. One-way ANOVA was used for statistical analysis (*n* = 3, *p < 0.05).

In the hippocampus, a low level of SM was observed in five-month-old *Hexa−/−Neu3−/−* mice compared to *WT* (Figure 4A). The level of TAG was significantly increased in *Hexa−/−Neu3−/−* mice compared to *WT* in the hippocampus (Figure 4B). Additionally, TAG was detected only in *Hexa−/−Neu3−/−* mice (Figure 4C). We found no significant changes in the levels of SE and Cer in the hippocampus of five-month-old *Hexa−/−Neu3−/−* mice (Supplementary Figure S3). A lower level of ST was also detected in the cortex of *Hexa−/−* and *Hexa−/−Neu3−/−* mice compared to *WT* mice but not in the hippocampus (Figures 5A,B).

### Histopathology and Immunohistochemistry Analysis

Previously, the accumulation of GM2 in the total brain of five-month-old *Hexa−/−Neu3−/−* mice had been demonstrated by HPTLC and mass spectrometric analysis (Seyrantepe et al., 2018). Here, we demonstrated that the number of GM2-containing neurons was significantly increased in the cortex and hippocampus of *Hexa−/−Neu3−/−* mice compared to *Hexa−/−* mice. No GM2 was detected in *WT* and *Neu3−/−* mice (Supplementary Figure S4).

**FIGURE 6:**
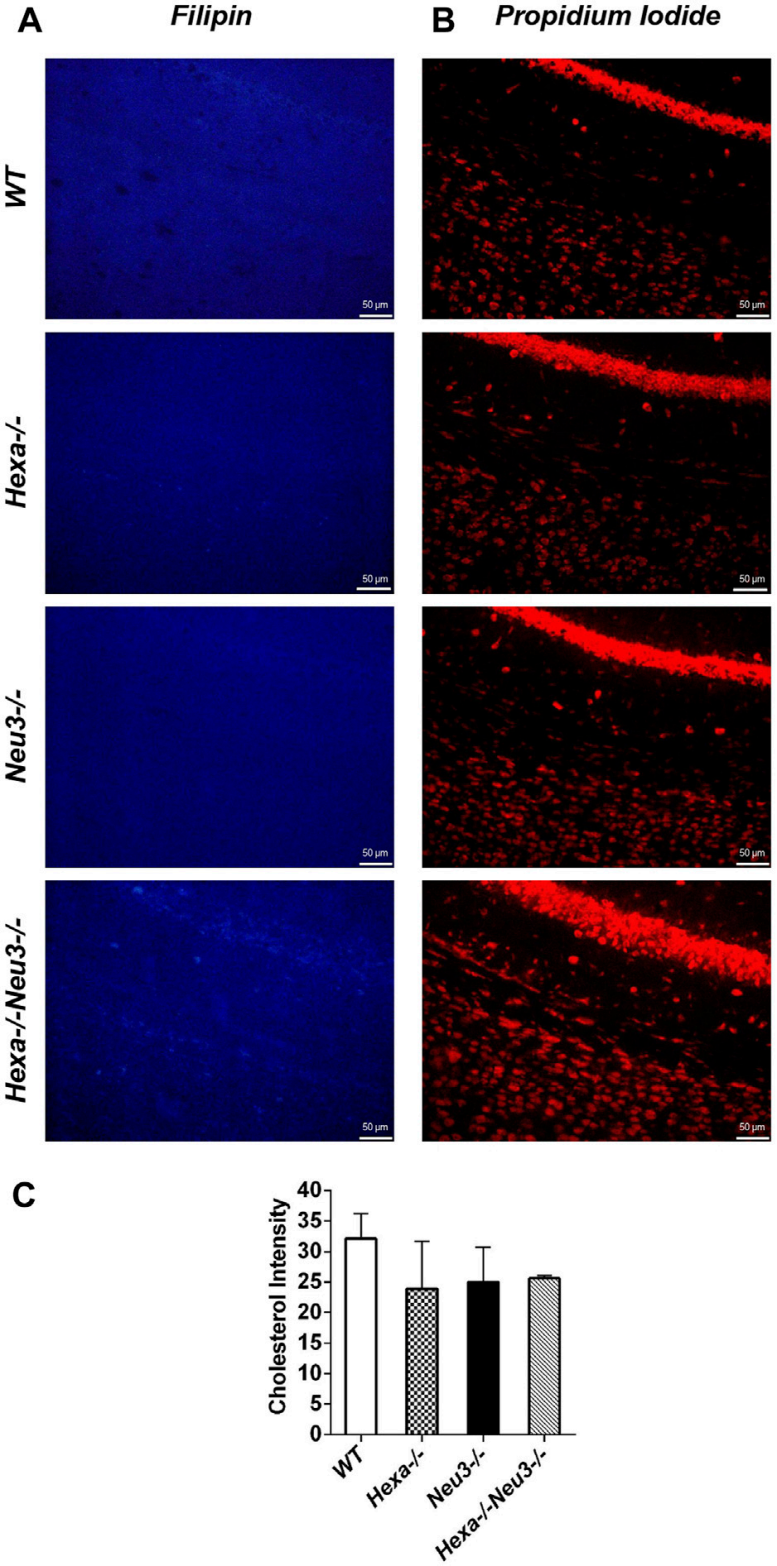
Detection of neuronal cholesterol in the cortex of five-month-old *WT*, *Hexa−/−*, *Neu3−/−*, and *Hexa−/−Neu3−/−* mice. The sections from the cortex of five-month-old *WT*, *Hexa−/−*, *Neu3−/−*, and *Hexa−/−Neu3−/−* mice were stained with filipin (blue) **(A)** and propidium iodide (red) **(B)**. The histograms represent the quantification of the cholesterol intensities in the cortex **(C)**. Scale bar = 50 µm. The data are represented as the mean ± SEM. One-way ANOVA was used for statistical analysis (*n* = 3).

**FIGURE 7:**
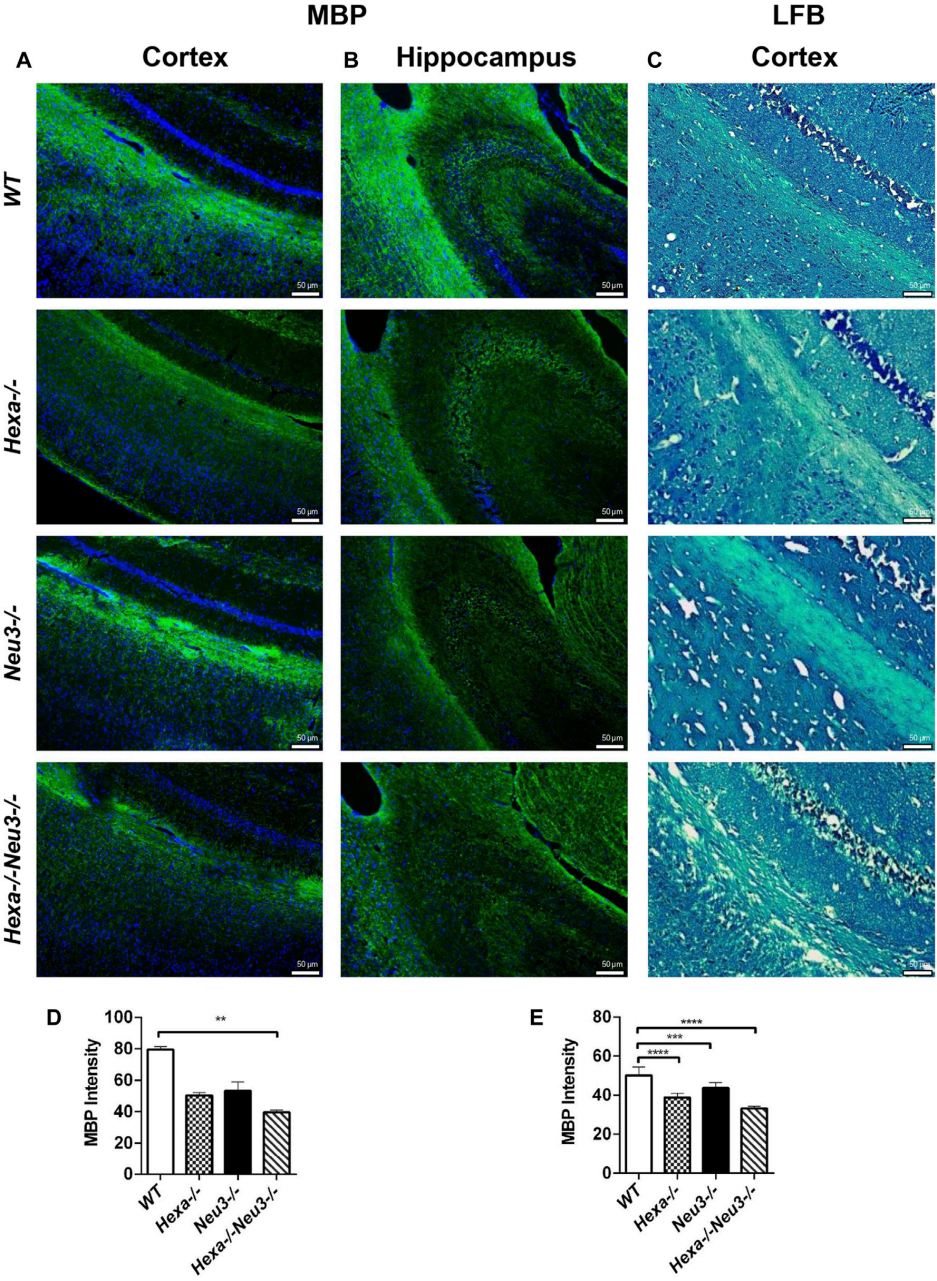
Immunohistochemical staining of myelin sheath in the cortex and hippocampus of five-month-old *WT*, *Hexa−/−*, *Neu3−/−*, and *Hexa−/−Neu3−/−* mice by anti-MBP and LFB staining. The sections from the cortex **(A)** and hippocampus **(B)** of five-month-old *WT*, *Hexa−/−*, *Neu3−/−*, and *Hexa−/−Neu3−/−* mice were stained with anti-MBP (green) and DAPI (blue) as well as sections from the cortex of **(C)** five-month-old *WT*, *Hexa−/−*, *Neu3−/−*, and *Hexa−/−Neu3−/−* mice were stained with the LFB. The histograms represent the quantification of the MBP intensities in the cortex **(D)** and hippocampus **(E)**, respectively. Scale bar = 50 µm. The data are represented as the mean ± SEM. One-way ANOVA was used for statistical analysis (*n* = 3, **p < 0.01, ***p < 0.005, and ****p < 0.001).

The levels of unesterified cholesterol in the cortex and hippocampus of *Hexa−/−Neu3−/−* mice were also analyzed using filipin dye and propidium iodide in parallel. We found that the cholesterol levels did not show any significant alterations between genotypes in both cortexes (Figure 6) and hippocampus (data not shown). To determine whether myelination is affected in *Hexa−/−Neu3−/−* brain, both anti-myelin basic protein (MBP) and luxol fast blue staining were performed. In the cortex, we detected a 34% reduction in the level of MBP-positive cells of *Hexa−/−Neu3−/−* mice compared to other genotypes indicating disrupted myelin (Figures 7A,D). Similarly, the hippocampal region showed significant reductions in the level of MBP-positive cells by 36% in *Hexa−/−*, 33% in *Neu3−/−*, and 50% in *Hexa−/−Neu3−/−* mice compared with that of *WT* (Figures 7B,E). Consistent with this finding, luxol fast blue staining revealed that myelin levels were reduced in the cortex of *Hexa−/−Neu3−/−* mice (Figure 7C).

**FIGURE 8:**
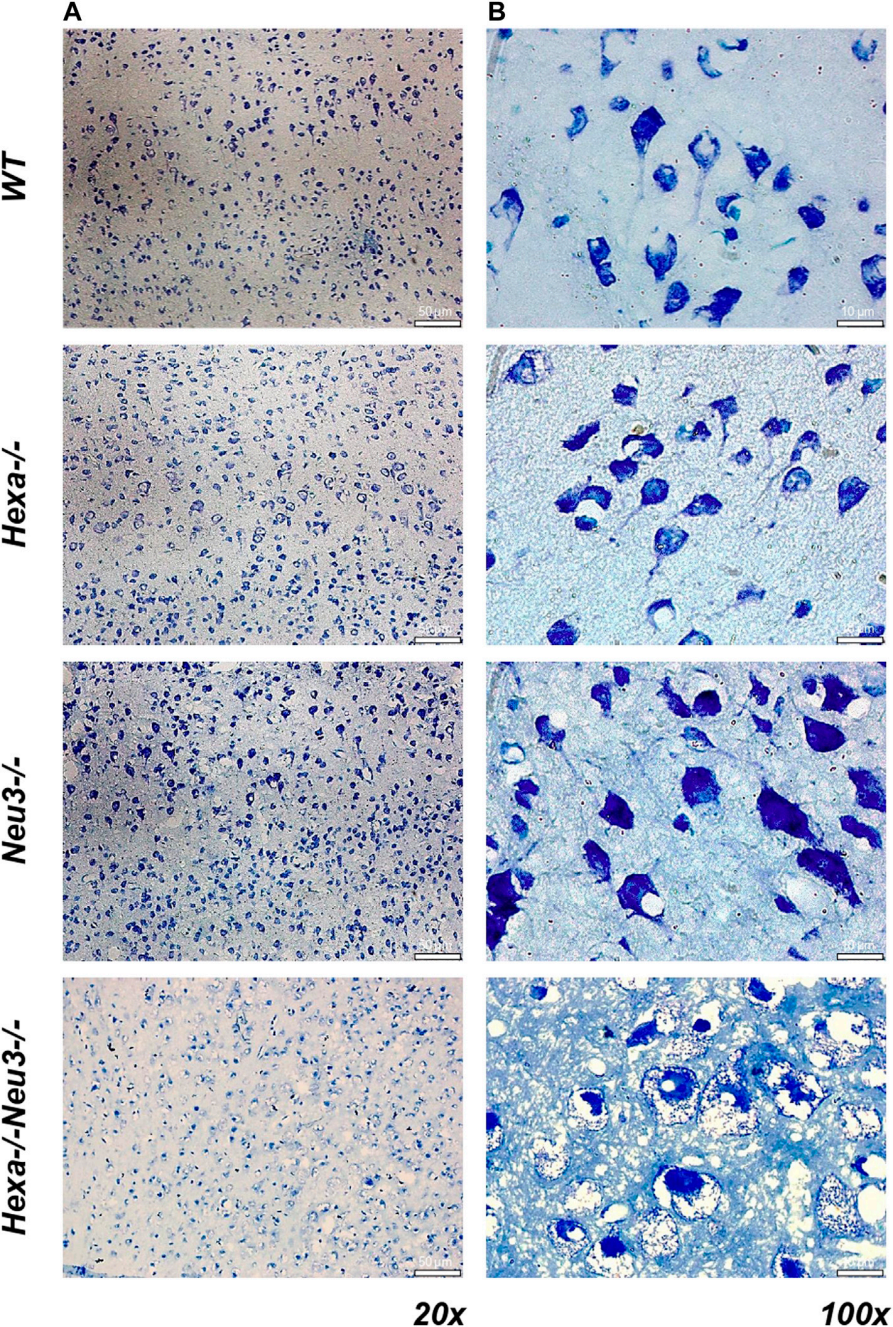
Toluidine blue staining in the cortex of five-month-old *WT*, *Hexa−/−*, *Neu3−/−*, and *Hexa−/−Neu3−/−* mice. The sections from the cortex of five-month-old *WT*, *Hexa−/−*, *Neu3−/−*, and *Hexa−/−Neu3−/−* mice were imaged at ×20 **(A)** and ×100 **(B)**. Scale bar = 50 and 10 µm (*n* = 3).

To analyze axonal degeneration, cortical sections from *WT*, *Hexa−/−*, *Neu3−/−*, and *Hexa−/−Neu3−/−* mice were stained with toluidine blue. We found numerous round large vacuoles in the cortical neurons of *Hexa−/−Neu3−/−* mice. Vacuoles were clearly seen at ×100 magnification for *Hexa−/−Neu3−/−* mice but not in other genotypes (Figure 8).

To characterize the level of glycoconjugates, five-month-old *WT*, *Hexa−/−*, *Neu3−/−*, and *Hexa−/−Neu3−/−*mice were stained with periodic acid and Schiff stain. We showed purple-stained inclusions in the cortex, hippocampus, and corpus callosum of *Hexa−/−Neu3−/−* mice neurons but not in other genotypes. Moreover, the perivascular macrophages shown in reddish-pink were seen in the cortex, hippocampus, and corpus callosum of *Hexa−/−Neu3−/−* mice compared to *WT*, *Hexa−/−*, and *Neu3−/−* mice (Supplementary Figure S5).

## Discussion

We previously generated *Hexa−/−Neu3−/−* mice that mimics the early-onset phenotype of TSD. In those mice, severe neurological abnormalities such as tremors and ataxia are observed. Similar to patients with TSD, *Hexa−/−Neu3−/−* mice also have growth impairment, short lifespan, progressive neurodegeneration, and behavioral defects. Primary accumulation of GM2, which is the hallmark of GM2 gangliosidosis, is observed in the *Hexa−/−Neu3−/−* mice model. In addition to GM2, secondary accumulation of glycosphingolipids such as GA2, GM3, and LacCer has been reported in five-month-old *Hexa−/− Neu3−/−* mouse brains (Seyrantepe et al., 2018). To address whether *Hexa−/−Neu3−/−* mice have other alterations in brain lipid, glycerophospholipids, glycerolipids, and sterol lipids were analyzed by shotgun lipidomics. To the best of our knowledge, this is the first study that shows lipid alterations in two brain regions from an early-onset TSD mice model.

Secondary accumulation of glycosphingolipids has been demonstrated in several LSDs. Patients with NP type A and C disease showed increased levels of GM2 ganglioside in the brain (Crocker, 1961; Cumings, 1962). Reduced levels of GD1a and GM1 along with increased levels of GD2, GD3, and GM3 gangliosides were also observed in the cerebral cortex and white matter of children who have Krabbe’s disease (Vanier and Svennerholm, 1975). As with other LSDs, brain specimens of Gaucher patients have increased levels of GM2 and GM3 gangliosides (Nilsson and Svennerholm, 1982). Similarly, secondary accumulation of GM2 and GM3 gangliosides have been reported in several MPS patients and MPS mice models (Constantopoulos and Dekaban, 1978). In addition, similar changes have been reported in NPC1- and NPC2-deficient mice models. Lipidomics analysis of NPC1-KO cells revealed the presence of several glycerophospholipids and other lipids in lysosomes (Tharkeshwar et al., 2017).

Deregulated sphingolipid metabolism leads to an abnormal sphingolipid profile causing several events associated with the pathogenesis of neurodegenerative diseases (Piccinini et al., 2010). Among the neurodegenerative disorders, altered lipid metabolism has been reported in both Alzheimer’s and Parkinson’s diseases. One study has shown reduced levels of PC, PI, and PE glycerophospholipids in the serum of Alzheimer’s patients (Kikuchi et al., 2002). Similarly, glycerophospholipids were also low in brain specimens of patients with Parkinson’s disease. Our shotgun lipidomics data revealed a reduced level of PC, PI, and PE glycerophospholipids in the cortex of *Hexa−/−Neu3−/−* mice, but only a decreased level of PC (Figure 2A) was detected in the hippocampus. Moreover, human brain tissues of Alzheimer’s disease and Parkinson’s disease had decreased levels of SM as well as increased levels of Cer (Fraser et al., 2010; Mesa-Herrera et al., 2019). Similarly, our data showed reduced levels of SM in both brain regions of *Hexa−/−Neu3−/−* mice. On the other hand, reduced levels of Cer were only observed in the cortex of *Hexa−/−Neu3−/−* mice but not in the hippocampus. The high levels of BMP, an isomer of PG, were reported in several LSDs such as NP, Gaucher, Fabry, and MPS I and II (Vanier 1983; Akgoc et al., 2015). In addition, a severe increase in BMP levels as a secondary accumulated lipid has been shown in brain samples of GM1 and GM2 gangliosidosis patients (Akgoc et al., 2015). We found a significantly elevated level of PG as an isomer of BMP only in the hippocampus of *Hexa−/−Neu3−/−* mice. Elevated levels of lyso-GM2 were shown in brain specimens from Tay–Sachs and Sandhoff disease patients. Altered phospholipid metabolism was also demonstrated in neuronal tissues of the Sandhoff disease mouse model. In addition to neurodegeneration, decreased levels of phospholipid synthesis are correlated with neuropathology in Sandhoff disease (Buccoliero et al., 2004). In Sandhoff mice, there was an increased level of lyso-GM2 in the brain and plasma compared to *WT* mice (Kodama et al., 2011). However, the levels of lyso-GM2 were not detectable in brain tissues of *Hexa−/−Neu3−/−* mouse.

NP disease is associated with the accumulation of cholesterol and other lipids in late endosomes/lysosomes (Walkley and Vanier., 2009). To check the secondary effects of GM2 accumulation on cholesterol metabolism, the amounts of unesterified cholesterol were visualized by filipin labeling. However, we found that the unesterified cholesterol level did not significantly change in the *Hexa−/−Neu3−/−* mouse brain*.*

The myelin sheath in the central nervous system is specifically formed in oligodendrocytes. In the previous study, we demonstrated that abnormal GM2 accumulation in the brain of *Hexa−/−Neu3−/−* mouse also causes the death of myelin-forming cells (Demir et al., 2020). Here, our shotgun lipidomics data showed a significant decrease in the level of SMs in both the cortex and hippocampus. The disruption of the myelin sheath was also confirmed by staining with anti-MBP and LFB. These results are consistent with other studies (Baek et al., 2009). Gene expression profile studies showed that the myelin basic protein gene was significantly lower in the cerebral cortex of GM2 gangliosidosis (Tay–Sachs and Sandhoff) patients than healthy individuals (Myerowitz et al., 2002). Axons of the cortical neurons in the five-month-old *Hexa−/−Neu3−/−* mice were significantly defected. Jackman et al. (2009) showed that sialylated gangliosides, especially GD1a and GT1b, are present on the axonal membrane and interact with the myelin-associated glycoprotein on the periaxonal surface to promote myelin sheath stability. Thus, we speculate that abnormal accumulation of GM2 as a sialic acid-containing ganglioside most likely leads to instability of the myelin sheath and axonal degeneration in the brain of the TSD mouse model.

Lipids are important components of biological membranes, and they are involved in several cellular processes including autophagy, apoptosis, and cellular stress (Singh et al., 2009). Recent work indicates the important role of lipids in autophagic flux. For instance, Cer has a regulatory role in the initiation, maturation, and termination of autophagy (Hernandez-Diaz and Soukup, 2020). On the other hand, conjugation of LC3 and PE on the phagophore is a key step of autophagy, and PE supplementation reduced accumulated endogenous substances including amyloid precursor protein and α-synuclein in cultured neurons and SGPL1-knockout mice, respectively (Mitroi et al., 2017). Moreover, it has been demonstrated that the accumulation of SM in endosomes damages the maturation of autophagosomes (Corcelle-Termeau et al., 2016). In addition, the protective effect of TAG biosynthesis in apoptosis has been shown (Li et al., 2018). In our study, we observed alterations in several lipids, such as PE, Cer, SM, and TAG, in brain regions of *Hexa−/−Neu3−/−* mice that might be linked to the dysfunctionality of lipid-related cellular pathways including autophagy and apoptosis. We suggest that further studies are necessary to clarify the relationship between altered lipid profiles and cellular events such as autophagy and apoptosis in the early-onset TSD mice model.

Taken together, our results clearly provide the first *in vivo* evidence that the brain of the TSD mouse model has additional alterations in lipid metabolism. Therefore, we suggest that having lipidome evaluation may lead to a detailed understanding of alterations in different lipid-driven mechanisms such as autophagy in TS and other lysosomal storage diseases.

## Data Availability

The original contributions presented in the study are included in the article/Supplementary Material; further inquiries can be directed to the corresponding author.

## Supplementary Material

Supplementary Figure S1 Lipidome analysis of LPA **(A)**, LPE **(B)**, LPS **(C)**, LPI **(D)**, and LPE O- **(E)** glycerophospholipids in the cortex of five-month-old *WT*, *Hexa−/−*, *Neu3−/−*, and *Hexa−/−Neu3−/−* mice. The data are represented as the mean ± SEM. One-way ANOVA was used for statistical analysis (*n*=3).

Supplementary Figure S2 Lipidome analysis of PE **(A)**, PE O- **(B)**, HexCer **(C)**, PI **(D)**, PA **(E)**, CL **(F)**, LPC O- **(G)**, LPS **(H)**, LPE O- **(I)**, and LPA **(J)** glycerophospholipids in the hippocampus of five-month-old *WT*, *Hexa−/−*, *Neu3−/−*, and *Hexa−/−Neu3−/−* mice. The data are represented as the mean ± SEM. One-way ANOVA was used for statistical analysis (*n*=3).

Supplementary Figure S3 Lipidome analysis of SE **(A)** and Cer **(B)** glycerolipids in the hippocampus of five-month-old *WT*, *Hexa−/−*, *Neu3−/−*, and *Hexa−/−Neu3−/−* mice. The data are represented as the mean ± SEM. One-way ANOVA was used for statistical analysis (*n*=3).

Supplementary Figure S4 mmunohistochemical analysis to detect GM2 ganglioside level in the cortex **(A)** and hippocampus **(B)** of five-month-old *WT*, *Hexa−/−*, *Neu3−/−*, and *Hexa−/−Neu3−/−* mice. The sections were labeled with anti-GM2 antibody (green) and DAPI (blue). The histograms represent the quantification of GM2 ganglioside in the cortex **(C)** and hippocampus **(D)**. Scale bar = 100 μm. The data are represented as the mean ± SEM. One-way ANOVA was used for statistical analysis (***p* < 0.01, ****p* < 0.005 and *****p* < 0.0001).

Supplementary Figure S5 Periodic acid–Schiff (PAS) staining of the cortex **(A)**, hippocampus **(B)**, and corpus callosum **(C)** regions of five-month-old *WT*, *Hexa−/−*, *Neu3−/−*, and *Hexa−/−Neu3−/−* mice. Sections from the cortex **(A)**, hippocampus **(B)**, and corpus callosum **(C)** regions of five-month-old *WT*, *Hexa−/−*, and *Neu3−/−* mice compared with five-month-old *Hexa−/−Neu3−/−* mice sections. Scale bar = 50 µm (*n*=3).
