# Supplementary figures and images for: Analysis of Brain Lipids in the Early-Onset Tay–Sachs Disease Mouse Model With the Combined Deficiency of β-Hexosaminidase A and Neuraminidase 3
Source: Front Mol Biosci. 2022 Aug 8;9:892248. doi: 10.3389/fmolb.2022.892248 (PMC9393265; doi:10.3389/fmolb.2022.892248)

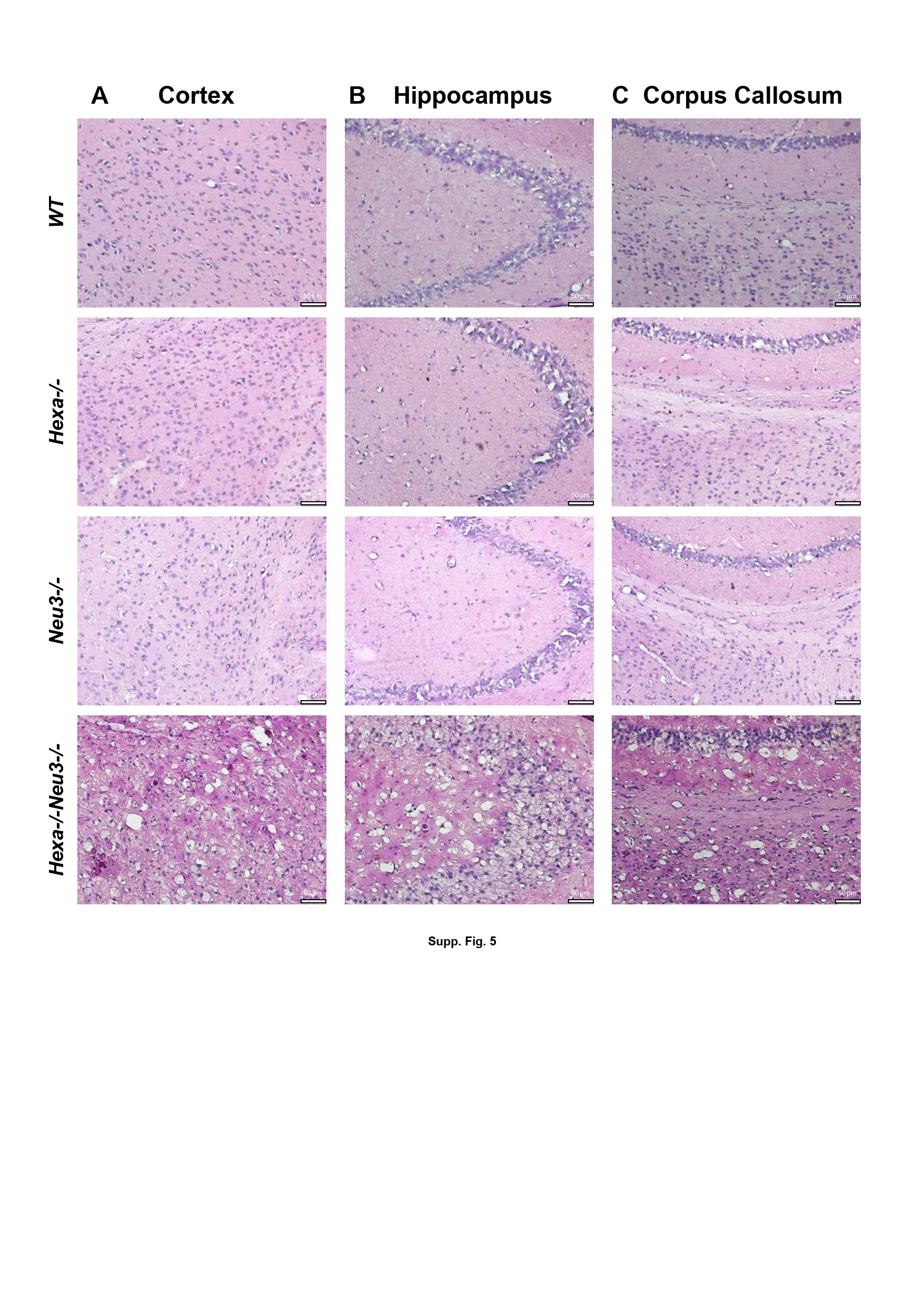

Supplement: Supplementary file 1 [file Image5.jpg]

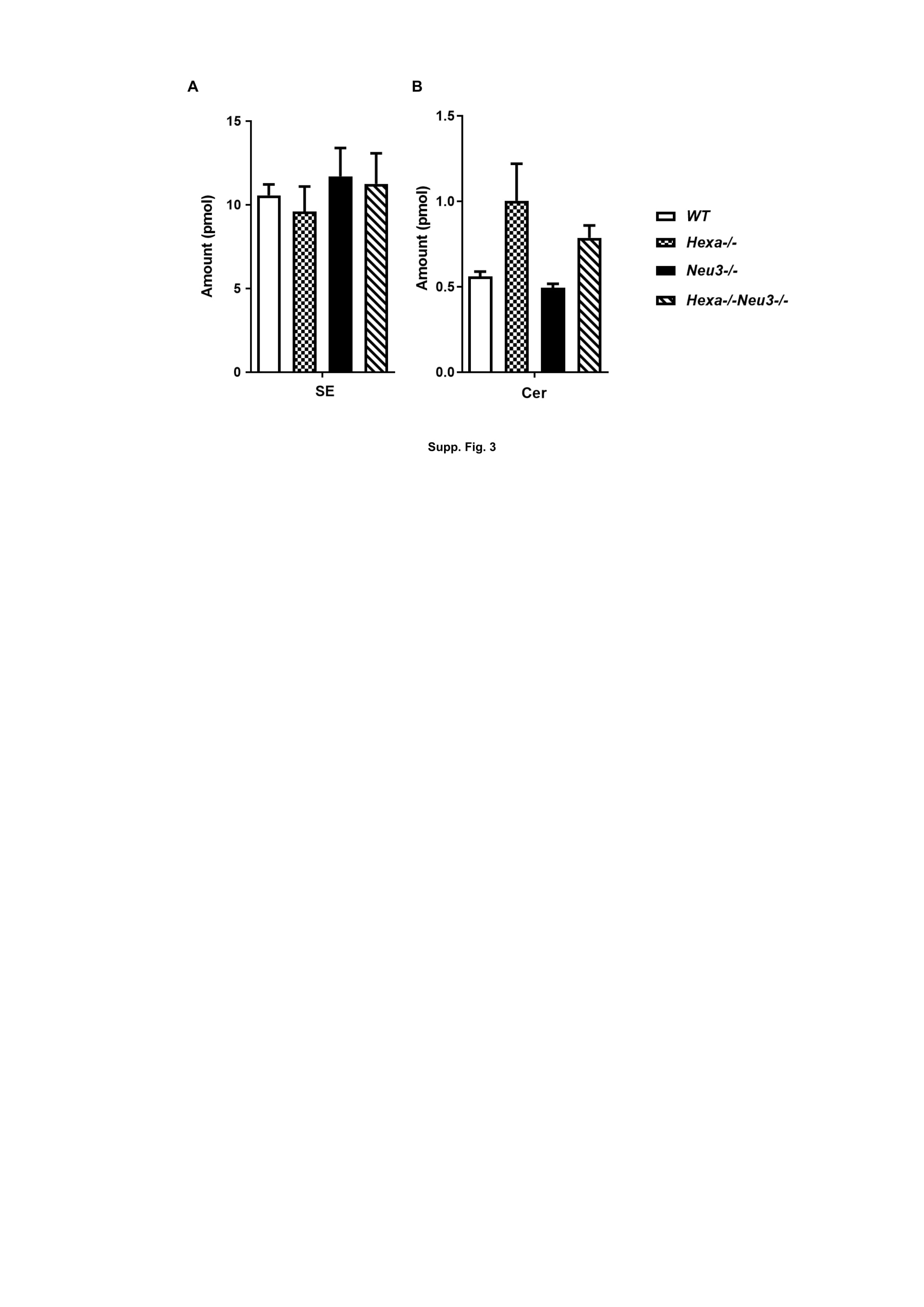

Supplement: Supplementary file 2 [file Image3.JPEG]

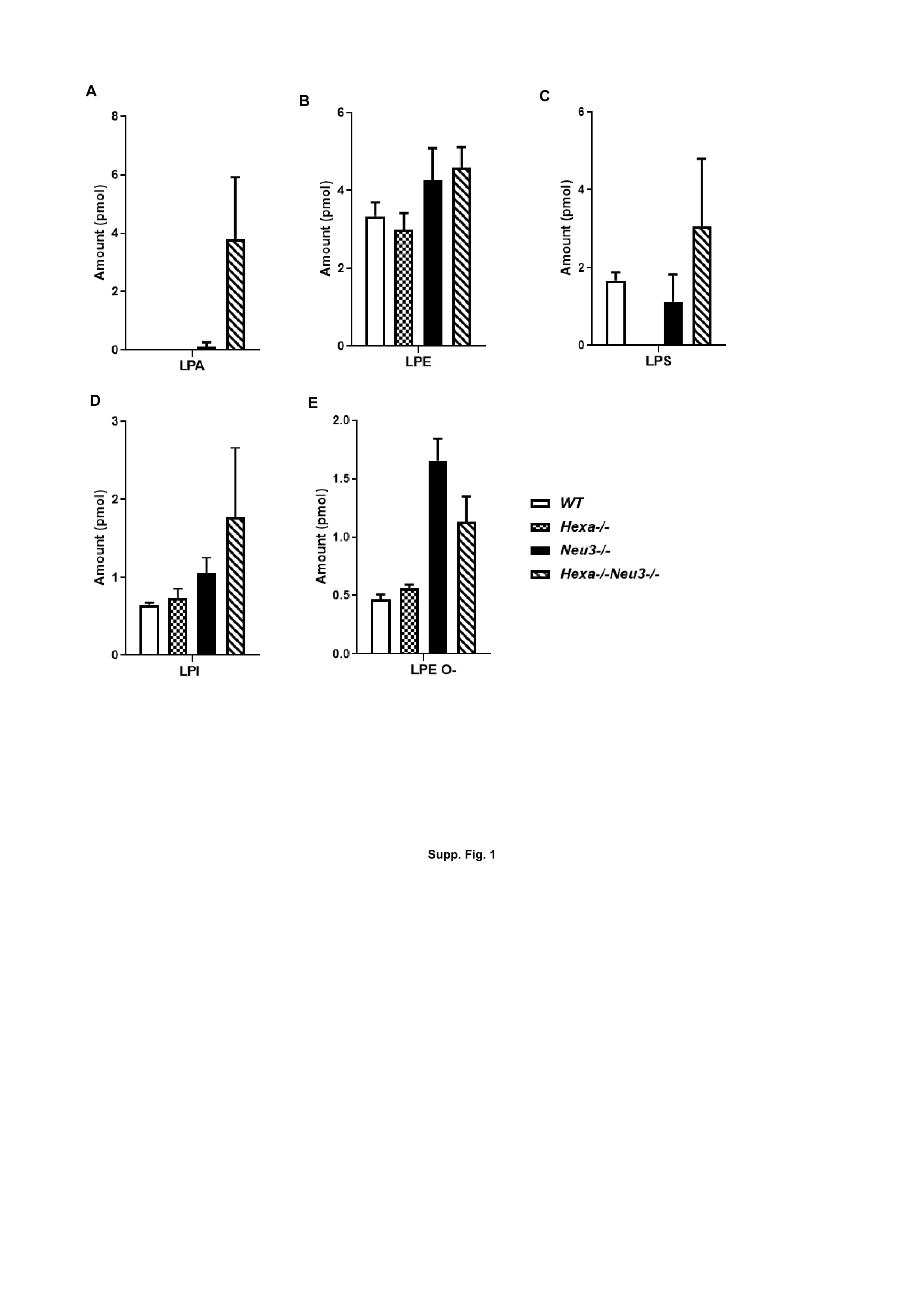

Supplement: Supplementary file 3 [file Image1.JPEG]

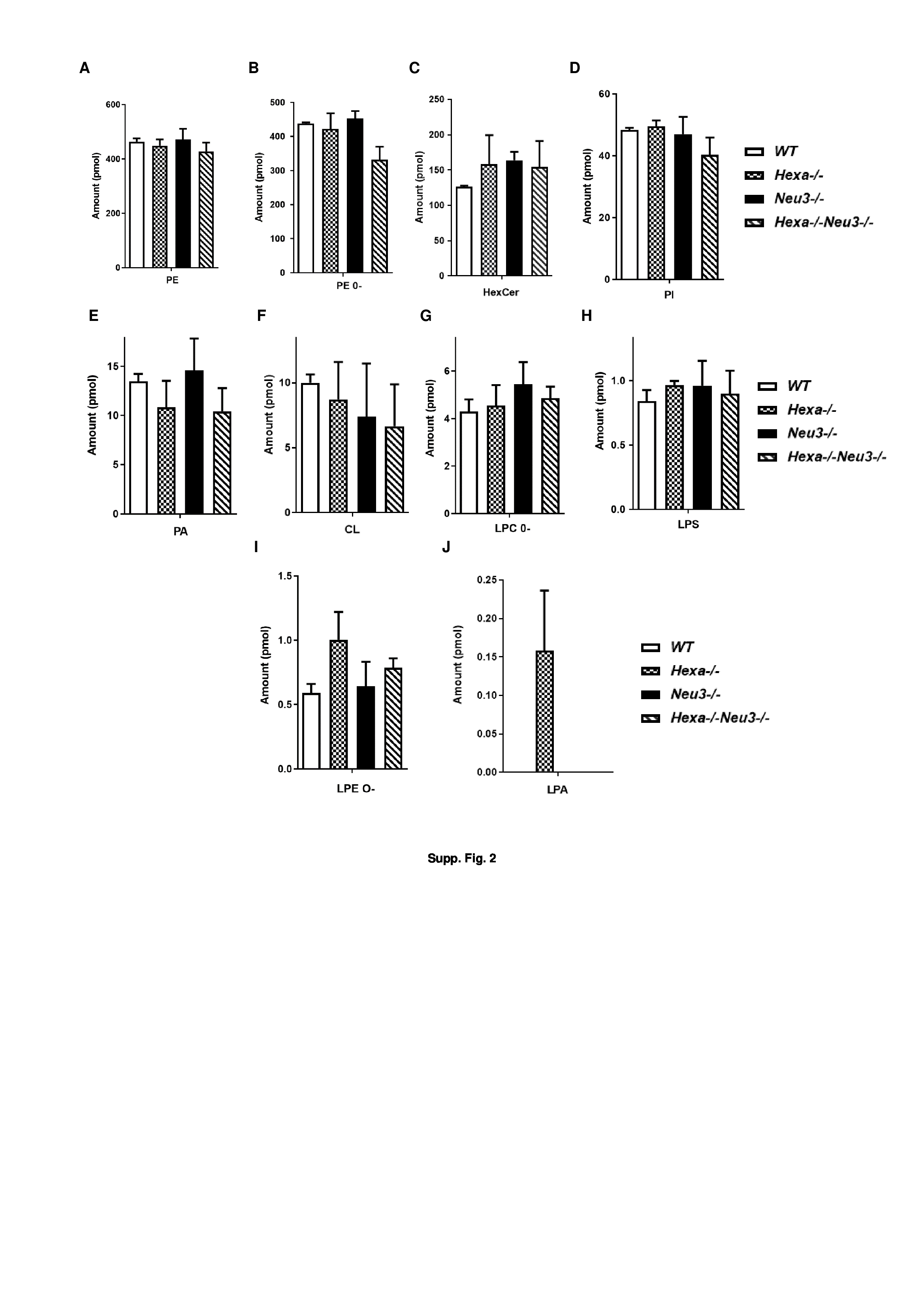

Supplement: Supplementary file 4 [file Image2.JPEG]

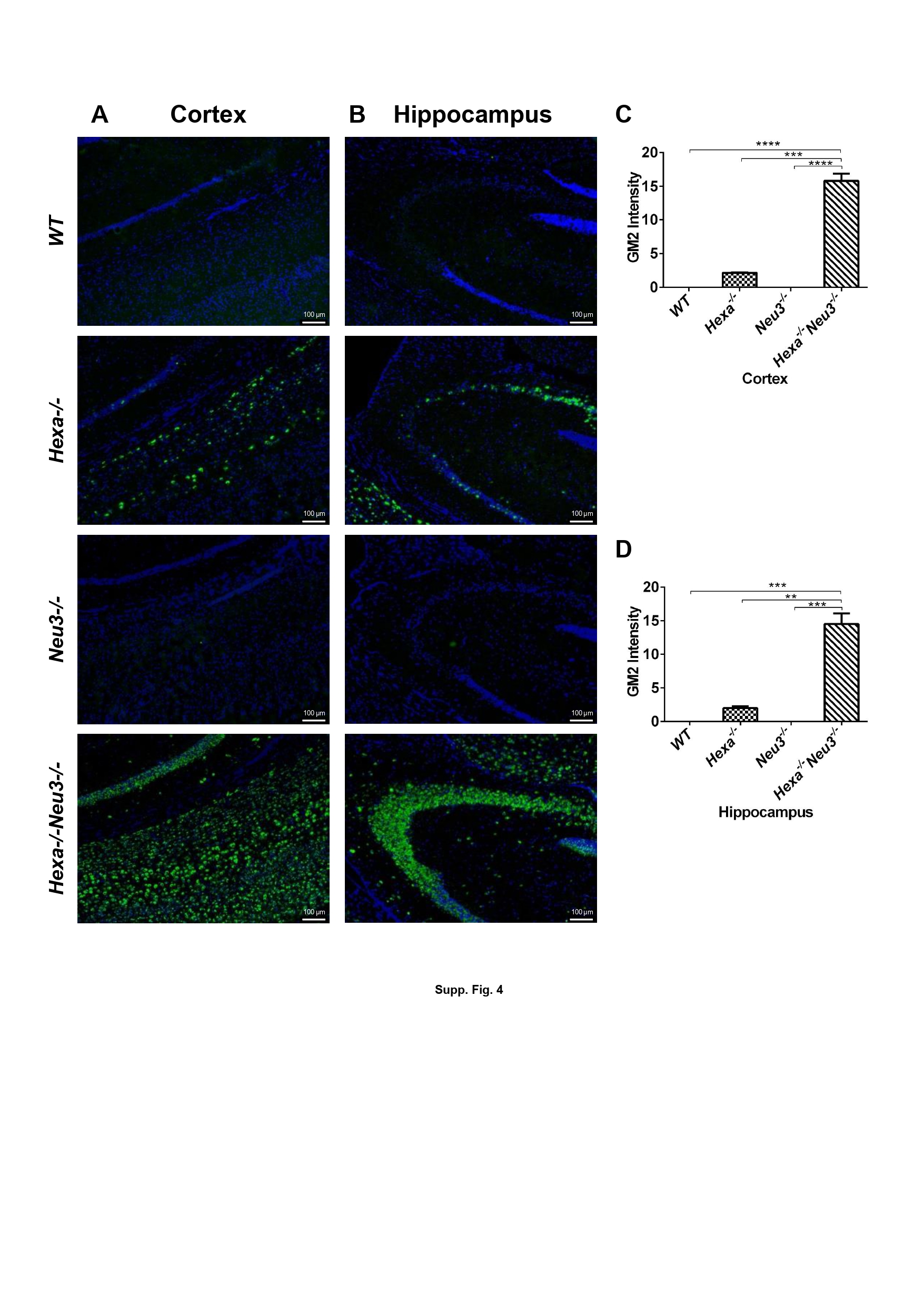

Supplement: Supplementary file 5 [file Image4.jpg]
